# FSSM: Frequency-Enhanced State Space Modeling with FFT-Based Two-Sided Non-Causal Convolution for Image Dehazing

**DOI:** 10.3390/jimaging12060260

**Published:** 2026-06-13

**Authors:** Li Zeng, Yinqing Huang

**Affiliations:** School of Mechatronics and Vehicle Engineering, Chongqing Jiaotong University, Chongqing 400074, China; zengli_sichuan@163.com

**Keywords:** image dehazing, state space model, FFT-based convolution, frequency-domain enhancement, UAV images

## Abstract

Image dehazing is a fundamental visual restoration task for improving visual perception under low-visibility weather conditions, especially in UAV-based remote sensing, traffic monitoring, and surveillance scenarios. Existing convolutional neural networks are effective in local feature extraction but remain limited in long-range dependency modeling, while Transformer-based methods improve global modeling at the cost of high computational complexity. To address these issues, this paper proposes an efficient image-dehazing framework termed FSSM, which integrates frequency-enhanced State Space Modeling with a hierarchical encoder–decoder architecture. Specifically, an FFT-based State Space Block (FFTSSB) is designed to reformulate state propagation as frequency-domain two-sided non-causal convolution, enabling efficient bidirectional global dependency modeling without explicit recursive scanning. Furthermore, a Frequency-Aware Discriminative Enhancement Block (FDEB) is introduced to enhance local textures, edges, and structural details through spatial gating and lightweight block-wise frequency modulation. Based on these two components, a Frequency-Aware State Interaction (FASI) block is constructed to progressively couple global state propagation and local frequency-aware enhancement. Experimental results on the HazyDet dataset demonstrate that FSSM achieves favorable restoration accuracy, structural consistency, and perceptual quality compared with representative dehazing methods. Ablation studies further validate the effectiveness of the proposed two-sided FFT-based state modeling, frequency-aware enhancement, and hierarchical multi-scale design.

## 1. Introduction

Image dehazing is a fundamental visual perception task under adverse weather conditions and plays a critical role in a wide range of applications, including autonomous driving, video surveillance, and remote sensing [1,2]. In such scenarios, image dehazing not only improves visual quality but also enhances the stability and robustness of downstream vision systems. Traditional dehazing methods are typically derived from the atmospheric scattering model and benefit from clear physical interpretability. Although these prior-based methods can achieve satisfactory results in relatively controlled scenarios, their performance often deteriorates in complex real-world scenes with non-uniform illumination and spatially varying haze, thereby limiting their generalization capability [3,4].

With the rapid development of deep learning, convolutional neural networks (CNNs) and vision Transformers have significantly advanced the performance of image dehazing. CNN-based methods mainly rely on sophisticated architectural designs and module innovations, such as residual connections [5], attention mechanisms [6], and large-kernel convolutions [7,8], to enhance feature representation. However, the inherently local receptive field of convolution limits the ability of CNNs to model long-range dependencies, often leading to insufficient global consistency and suboptimal detail recovery [9]. In contrast, Transformer-based models introduce self-attention mechanisms to capture global contextual information and have achieved remarkable progress in image dehazing [10,11]. Nevertheless, the quadratic computational complexity of self-attention with respect to input resolution severely restricts their efficiency, especially for high-resolution images and real-time or mobile deployment scenarios [12,13].

Recently, State Space Models (SSMs) [14,15] have attracted considerable attention because of their ability to model long-range dependencies with linear computational complexity. By parameterizing sequence dynamics through exponential kernels, SSMs have demonstrated impressive performance in time-series modeling, natural language processing, and signal processing tasks. Notably, the improved SSM architecture Mamba [16,17] introduces a selective scanning mechanism that enables the model to efficiently retain task-relevant information while suppressing irrelevant components, offering an attractive balance between modeling capacity and computational efficiency. These properties motivate the exploration of SSMs for image restoration tasks, including image dehazing, where effective non-local modeling is crucial.

However, directly applying SSMs to image dehazing poses several challenges. Naïve adaptations often introduce redundant computations and directional bias, particularly when modeling bidirectional spatial dependencies inherent in images [18,19,20]. Moreover, existing SSM formulations are not explicitly designed to exploit frequency-domain characteristics, which are highly informative for haze-related degradation patterns.

To address these limitations, this paper proposes a novel and efficient image-dehazing framework termed FSSM (Frequency-Enhanced State Space Model). The proposed approach integrates the continuous dynamic modeling capability of State Space Models with frequency-domain enhancement mechanisms, enabling collaborative optimization of global dependency modeling and local detail restoration. Specifically, the main contributions of this work are summarized as follows:

- We propose an FFT-based State Space Block (FFTSSB), which performs two-sided non-causal convolution in the frequency domain to replace conventional recursive state propagation. This design enables implicit state propagation with reduced computational redundancy, facilitating efficient global dependency modeling while preserving physical interpretability.
- We design a Frequency-Aware State Interaction (FASI) Block, which tightly couples FFTSSB with the Frequency-Aware Discriminative Enhancement Block (FDEB). This unified spatial–frequency modeling unit enhances texture and edge restoration and improves robustness to complex haze structures.
- We construct a hierarchical multi-scale encoder–decoder framework with skip connections, allowing effective cross-scale feature interaction and fusion. This design significantly improves detail recovery and structural consistency in dehazed images.
- Extensive experiments on the HazyDet dataset demonstrate that FSSM achieves favorable quantitative and qualitative dehazing performance, with improvements in PSNR and SSIM over representative comparison methods. Ablation studies further verify the effectiveness of the proposed FFTSSB and FDEB modules in enhancing global structural consistency and local detail restoration.

## 2. Related Work

### 2.1. Traditional Image-Dehazing Methods

Traditional image-dehazing methods are mainly based on physical models or handcrafted statistical priors. The dark channel prior (DCP) proposed by He et al. [4] assumes that at least one color channel in non-sky regions has very low intensity, and has become a classical approach for single-image dehazing. Zhu et al. proposed the color attenuation prior (CAP) [21], which estimates scene depth via linear regression to recover haze-free images. In addition, Berman et al. introduced the non-local dehazing (NLD) method [22], which reconstructs the transmission map by exploiting color clustering properties in natural images.

Although these prior-based methods exhibit strong physical interpretability and can produce visually pleasing results under certain conditions, they generally rely on idealized assumptions. As a result, their performance often degrades in complex environments, such as nighttime scenes, non-uniform haze, or strong illumination conditions [23]. Moreover, due to the lack of adaptive learning capability, these methods struggle to capture fine-grained texture details, frequently leading to over-smoothed results or color distortions, which limits their practical applicability and generalization ability.

### 2.2. Deep Learning-Based Dehazing Methods

Deep learning techniques have significantly advanced the development of image dehazing. AOD-Net [24] was among the first to embed the atmospheric scattering model into a neural network, enabling end-to-end haze removal. FFA-Net [25] introduced feature attention mechanisms to enhance texture restoration, while MixDehaze-Net [26] adopted multi-scale dense architectures to improve detail reconstruction. OKNet [27] proposed an omni-kernel module that integrates global, large-scale, and local branches, achieving effective multi-scale feature learning for image dehazing, desnowing, and deblurring tasks.

Furthermore, DWTMA-Net [28] combined discrete wavelet transform (DWT) with multi-dimensional attention mechanisms to jointly recover spatial- and frequency-domain features, significantly improving detail fidelity and color consistency. Transformer-based methods, such as MB-TaylorFormer [29], U^2^-Former [30], and SelfPromer [31] leverage self-attention mechanisms to capture long-range dependencies and have achieved strong dehazing performance. However, their high computational complexity makes them difficult to deploy in real-time systems. Recently, hybrid architectures, including Trinity-Net [32] and Two-Branch Image Dehazing [33], have attempted to combine the local representation capability of CNNs with the global modeling strength of Transformers, achieving a more favorable trade-off between performance and efficiency.

More recently, researchers have further explored real-world and diverse haze removal from the perspectives of domain adaptation, generative modeling, degradation diversity, and lightweight deployment. UCL-Dehaze [34] introduced an unsupervised contrastive learning paradigm to alleviate the domain gap between synthetic and real hazy images. Dehaze-RetinexGAN [35] exploited Retinex-based decomposition and generative adversarial learning for real-world dehazing with unpaired data. MDST-Dehaze [36] considered the diversity of haze distributions across different domains and employed multi-domain haze-style transfer to improve robustness under heterogeneous haze conditions. From the perspective of generative restoration, PRDiff-Dehaze [37] adopted a progressive refinement diffusion framework to handle non-homogeneous haze degradation. Meanwhile, WaveLiteDehaze-Network [38] explored lightweight wavelet-domain processing for real-time dehazing with a small number of parameters.

In addition, recent Transformer-based and State Space Model-based image restoration methods have shown strong potential for long-range dependency modeling. Methods such as DehazeFormer, Restormer, UVM-Net, and Mamba-style image restoration networks provide useful references for improving global feature interaction and restoration quality. However, attention-based Transformer models usually introduce relatively high computational cost when processing high-resolution images, while many SSM/Mamba-style methods still rely on directional scanning or do not explicitly exploit haze-related frequency-domain characteristics. In contrast, the proposed FSSM reformulates state propagation as FFT-based two-sided non-causal convolution and further integrates frequency-aware discriminative enhancement. This design aims to jointly improve global structural consistency and local detail restoration while maintaining computational efficiency.

Overall, these recent studies indicate that image dehazing is moving toward real-world adaptability, degradation-aware modeling, generative restoration, long-range dependency modeling, and efficient deployment. Nevertheless, most existing methods still face challenges in simultaneously modeling long-range structural dependencies, enhancing frequency-domain details, and maintaining computational efficiency. This motivates us to explore a frequency-enhanced State-Space Modeling framework that can jointly improve global structural consistency and local detail restoration while preserving efficiency.

### 2.3. State Space Models for Image Dehazing

State Space Models (SSMs) have recently demonstrated great potential for long-range dependency modeling with linear or near-linear computational complexity, particularly in natural language processing tasks. The improved SSM architecture Mamba [39] introduces a selective scanning mechanism that adaptively allocates memory to task-relevant features, enabling efficient dynamic sequence modeling and achieving remarkable performance gains in various NLP and signal processing applications.

In the vision domain, researchers have begun to explore the application of SSMs to image restoration and dehazing. VMamba and Wave-MambaAD model implicit global dependencies in the spatial domain through parameterized convolutional kernels, improving structural consistency and dehazing quality. UVM-Net [40] proposed a bidirectional state-space module (Bi-SSM) that integrates convolutional local feature extraction with global modeling capability of SSMs, achieving a balance between performance and computational complexity for single-image dehazing. Laplace-Mamba [41] further combines Laplacian pyramid decomposition with a Mamba–CNN hybrid architecture to separately process low-frequency global structures and high-frequency details, significantly enhancing image hierarchy and edge sharpness.

Despite these advances, existing SSM-based dehazing methods still suffer from relatively high computational overhead and directional redundancy. This motivates the development of more efficient spatial–frequency joint modeling mechanisms to further improve dehazing performance and deployment efficiency.

## 3. Methodology

### 3.1. Notation

To improve mathematical clarity and readability, we adopt unified notation conventions throughout this paper. Bold uppercase symbols, such as I, X, and F, denote image or feature tensors. Bold uppercase symbols such as A, B, C, and D denote learnable matrices in the state-space formulation. Bold lowercase symbols, such as x, h, and y, denote vectors or feature sequences. Lowercase italic symbols, such as *t*, *H*, *W*, *C*, *R*, and λ, denote scalar variables. Calligraphic symbols, such as F(·), $F^{-1}\left(\cdot \right)$, and N(·), denote operators or network mappings. The notation ⊙ denotes element-wise multiplication.

### 3.2. Overall Architecture

This paper proposes a frequency-enhanced State Space Modeling framework based on FFT-based two-sided non-causal convolution, termed FSSM, for efficient single-image dehazing. The core idea is to leverage frequency-domain convolution to efficiently model global dependencies while enhancing state-space representations, thereby achieving high-quality image dehazing.

**Figure 1:**
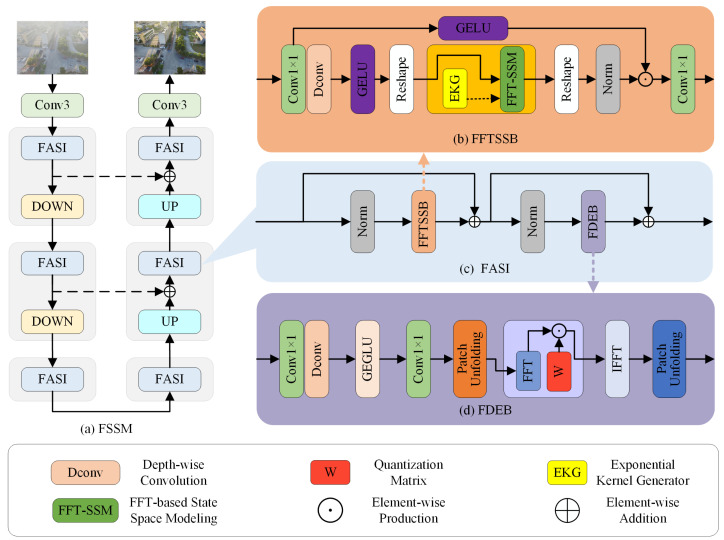
Overall architecture of the proposed FSSM framework. (**a**) The overall FSSM architecture; (**b**) FFT-Based State Space Block (FFTSSB); (**c**) Frequency-Aware State Interaction (FASI) block; (**d**) Frequency-Aware Discriminative Enhancement Block (FDEB).

As illustrated in Figure 1, the proposed FSSM adopts a hierarchical encoder–decoder architecture [42]. Given a hazy input image $I_{\mathrm{hazy}}\in R^{H\times W\times 3}$, a 3×3 convolution is first applied to extract shallow features $F_{s}\in R^{H\times W\times C}$, where H×W denotes the spatial resolution and *C* represents the number of feature channels. The shallow features are then fed into a three-level symmetric encoder–decoder network, in which each encoder and decoder stage consists of stacked Frequency-Aware State Interaction (FASI) blocks for spatial–frequency feature modeling at different scales.

For the encoder and decoder at the *l*-th level, the input features are progressively transformed through stacked FASI blocks, generating intermediate representations $F_{\mathrm{enc}}^{l}$ and $F_{\mathrm{dec}}^{l}$, where $F_{\mathrm{enc}}^{l},F_{\mathrm{dec}}^{l}\in R^{\frac{H}{2^{l-1}}\times \frac{W}{2^{l-1}}\times C_{l}}$ and l=1,2,3. Spatial resolution changes and channel alignment are achieved via 1×1 convolutions combined with bilinear interpolation for downsampling and upsampling, respectively. Skip connections are introduced between corresponding encoder and decoder stages to facilitate the fusion of shallow fine-grained details and deep semantic information, thereby preserving structural boundaries and texture details.

Finally, a 3×3 convolution is applied to the output features of the last decoder stage to predict the residual image $R\in R^{H\times W\times 3}$. The residual image is defined as:

(1) $$R=N(I_{\mathrm{hazy}}),$$

where N(·) denotes the proposed FSSM dehazing network. The final dehazed image is obtained by adding the predicted residual image to the hazy input:

(2) $$I_{\mathrm{dehaze}}=I_{\mathrm{hazy}}+R.$$

This residual formulation is also explicitly illustrated in Figure 1a, where the network output is first treated as the residual image and then added to the input hazy image to generate the final dehazed result.

### 3.3. State Space Modeling and Its Extensions

#### 3.3.1. State Space Model Foundation

State Space Models (SSMs) provide a general framework for modeling dynamic systems and have been widely applied in time-series prediction and signal processing. The relationship among the input, latent state, and output can be formulated as:

(3) $$h^{\prime }\left(t\right)=Ah\left(t\right)+Bx\left(t\right),\;y\left(t\right)=Ch\left(t\right)+Dx\left(t\right),$$

where x(t), h(t), and y(t) denote the input, latent state, and output at continuous time *t*, respectively. A, B, C, and D are learnable system matrices.

By applying zero-order hold (ZOH) discretization, the continuous formulation can be rewritten as:

(4) $$h_{t}=\bar{A}h_{t-1}+\bar{B}x_{t},\;y_{t}=Ch_{t}+Dx_{t},$$

where $x_{t}$, $h_{t}$, and $y_{t}$ denote the discretized input, hidden state, and output at index *t*, respectively. $\bar{A}$ and $\bar{B}$ are the discretized state transition and input matrices.

Mamba [16] introduces a selective scanning mechanism (S6) that enables linear-complexity sequence modeling by adaptively controlling information flow. However, when applied to vision tasks, two major limitations arise: (1) unidirectional state propagation restricts the modeling of bidirectional spatial dependencies; and (2) explicit recursive updates incur considerable computational overhead for high-resolution feature maps.

#### 3.3.2. FFT-Based State Space Block (FFTSSB)

To efficiently model long-range dependencies in vision tasks, we propose an FFT-Based State Space Block (FFTSSB). The core idea of this block is to reformulate state-space propagation as a convolution process and then implement the resulting two-sided non-causal convolution in the frequency domain, thereby enabling efficient implicit state propagation with reduced computational overhead.

For a discrete State Space Model, the hidden-state update can be written as a weighted summation over historical inputs. Under a zero-initial-state assumption, the state response can be unfolded as:

(5) $$h_{t}=\sum_{\tau =0}^{t}{\bar{A}}^{\;t-\tau }\bar{B}x_{\tau },$$

where $\bar{A}$ and $\bar{B}$ denote the discretized state transition and input matrices, respectively, and $x_{\tau }$ denotes the input feature sequence at index τ. This formulation indicates that the State-Space Model can be interpreted as a discrete convolution process between the input sequence and the system impulse response.

Accordingly, the impulse response kernel can be defined as:

(6) $$k\left[t-\tau \right]={\bar{A}}^{\;t-\tau }\bar{B},$$

where k[t−τ] denotes the state-space impulse response kernel corresponding to the relative index t−τ. The state update can then be uniformly rewritten as:

(7) $$h_{t}=\sum_{\tau =0}^{t}k\left[t-\tau \right]x_{\tau }.$$

To enable effective long-range dependency modeling with a compact parameterization, we adopt an exponential kernel generator (EKG) to parameterize the impulse response. Specifically, following the exponential kernel parameterization strategy in prior State Space Models [43], the *i*-th exponential basis function is defined as:

(8) $$\phi_{i}\left(t\right)=e^{-a_{i}t},\;a_{i}=\mathrm{softplus}\left({\tilde{a}}_{i}\right)+\epsilon ,\;t\in \left\{0,1,\ldots ,T\right\}.$$

where $a_{i}$ denotes the learnable decay rate, ${\tilde{a}}_{i}$ is the learnable parameter before activation, ϵ is a small positive constant for numerical stability, and *T* denotes the maximum sequence length.

Based on these exponential bases, the channel-wise single-sided impulse response for the *c*-th channel is constructed as:

(9) $$h^{\left(c\right)}\left[t\right]=\sum_{i=1}^{S}w_{i}^{\left(c\right)}e^{-a_{i}t},$$

where $h^{\left(c\right)}\left[t\right]$ denotes the single-sided impulse response at index *t* in the *c*-th channel, $w_{i}^{\left(c\right)}$ denotes the linear combination coefficient of the *i*-th basis in the *c*-th channel, and *S* is the number of basis functions. This parameterization corresponds to the exponential decay response of a continuous-time linear system and provides an interpretable and flexible mechanism for modeling dependencies at different scales.

However, conventional State Space Models generally adopt a causal propagation manner and only utilize historical information for state updating, which is insufficient for capturing the bidirectional spatial dependencies inherent in images. To address this limitation, we further extend the single-sided kernel to a symmetric two-sided non-causal kernel through mirror expansion:

(10) $$k^{\left(c\right)}\left[\tau \right]=\left\{\begin{array}{cc} h^{\left(c\right)}\left[-\tau \right], & \tau <0,\\ h^{\left(c\right)}\left[\tau \right], & \tau \geq 0. \end{array}\right.$$

where $k^{\left(c\right)}\left[\tau \right]$ denotes the two-sided non-causal convolution kernel in the *c*-th channel, and τ represents the relative spatial offset.

Compared with one-sided FFT-based convolution, which only aggregates information from one direction, the proposed two-sided non-causal convolution incorporates both preceding and following spatial contexts. This is more consistent with the non-causal nature of image restoration, where the recovery of each pixel or patch should depend on surrounding structures from multiple directions rather than only historical positions. Therefore, the two-sided design reduces directional bias and enhances global structural consistency, which is particularly important for UAV dehazing scenes with large sky regions, long-range structures, and spatially non-uniform haze.

Given the input feature sequence $x\left[t\right]\in R^{C}$, where $x^{\left(c\right)}\left[t\right]$ denotes the feature at position *t* in the *c*-th channel, the corresponding convolution output is expressed as:

(11) $$y^{\left(c\right)}\left[t\right]=\sum_{\tau =-R}^{R}k^{\left(c\right)}\left[\tau \right]\;x^{\left(c\right)}\left[t-\tau \right],$$

where *R* denotes the convolution radius and $y^{\left(c\right)}\left[t\right]$ denotes the output feature at position *t* in the *c*-th channel. In this way, FFTSSB is able to simultaneously capture forward and backward spatial dependencies, thereby enhancing global structure modeling capability.

To further improve computational efficiency, the above convolution is implemented in the frequency domain according to the convolution theorem:

(12) $$Y^{\left(c\right)}=F\left\{x^{\left(c\right)}\right\}⊙F\left\{k^{\left(c\right)}\right\},\;y^{\left(c\right)}=F^{-1}\left\{Y^{\left(c\right)}\right\},$$

where F(·) and $F^{-1}\left(\cdot \right)$ denote the Fourier transform and inverse Fourier transform, respectively, $Y^{\left(c\right)}$ denotes the frequency-domain representation of the convolution result, and ⊙ represents element-wise multiplication. By performing convolution in the frequency domain, FFTSSB avoids explicit recursive state updates and significantly reduces the computational burden while preserving strong global modeling capability.

From a theoretical perspective, the proposed FFTSSB can be viewed as a convolutional realization of the discrete solution to the state-space equations. Through the interpretable exponential kernel parameterization provided by the EKG and the symmetric two-sided non-causal extension, FFTSSB enables efficient bidirectional state propagation in the frequency domain. Compared with the unidirectional modeling strategies adopted in S4 [43] and Hyena [44], the proposed design is more suitable for image dehazing, where jointly modeling long-range structural dependencies and spatially distributed degradation patterns is crucial. Moreover, FFTSSB provides globally coherent feature priors for the subsequent Frequency-Aware State Interaction (FASI) block, facilitating the effective integration of global structure modeling and local detail restoration.

#### 3.3.3. Frequency-Aware Discriminative Enhancement Block (FDEB)

Although State Space Models (SSMs) are effective in modeling long-range dependencies and global structures, accurate recovery of local textures and edge details remains equally important for image restoration tasks such as image dehazing. To compensate for the limited local discriminative modeling capability of SSM-based global representations, we introduce a Frequency-Aware Discriminative Enhancement Block (FDEB), which is designed to refine the features produced by the FFT-Based State Space Block (FFTSSB) through explicit local spatial filtering and lightweight frequency-domain modulation.

In deep neural networks, feed-forward structures are commonly used for nonlinear transformation and local feature enhancement, and have been shown to play an important role in image restoration tasks [45]. Recent studies further indicate that introducing frequency-domain operations into feed-forward networks can improve restoration quality by selectively enhancing informative spectral components. For example, FFTformer [46] employs frequency-domain feature modulation to strengthen texture and detail reconstruction. However, directly performing Fast Fourier Transform (FFT) on high-dimensional intermediate feature maps usually incurs considerable computational overhead, especially when the feature resolution and channel number are large.

To address this issue, FDEB adopts a lightweight spatial-frequency collaborative design. Instead of applying FFT directly to the original high-dimensional feature map, FDEB first performs local spatial screening through gated transformation and then conducts block-wise frequency-domain modulation on the filtered features. In this way, local discriminative enhancement can be achieved while keeping the computational cost under control.

Given an input feature tensor $X\in R^{H\times W\times C}$, FDEB first maps it into a higher-dimensional hidden space through a 1×1 convolution:

(13) $$X_{1}={\mathrm{Conv}}_{1\times 1}\left(X\right),$$

where $X_{1}$ denotes the projected hidden feature.

Then, a depth-wise convolution is applied to capture local spatial contextual information, and the resulting features are evenly split into two branches along the channel dimension:

(14) $$X_{2},X_{3}=\mathrm{Split}\left(\mathrm{DConv}\left(X_{1}\right)\right),$$

where DConv(·) denotes the depth-wise convolution operation, and Split(·) represents channel-wise splitting. $X_{2}$ and $X_{3}$ denote two feature branches generated from $X_{1}$ with the same spatial resolution. The former is activated by GELU to generate a nonlinear gating signal, while the latter provides local spatial features for modulation.

A gated activation mechanism is then introduced to perform local feature selection:

(15) $$X_{4}=\mathrm{GELU}\left(X_{2}\right)⊙X_{3},$$

where GELU(·) is the Gaussian Error Linear Unit and ⊙ represents element-wise multiplication.

After local gating, a 1×1 convolution is used to project the feature back to the original channel dimension, yielding the spatial feature for subsequent frequency enhancement:

(16) $$X_{s}={\mathrm{Conv}}_{1\times 1}\left(X_{4}\right),$$

where $X_{s}$ denotes the spatially gated feature after channel projection.

To avoid the computational burden caused by directly applying frequency transformation to the entire feature map, FDEB performs block-wise frequency modeling. Specifically, the spatial feature $X_{s}$ is first partitioned into a set of local patches:

(17) $$X_{p}=P\left(X_{s}\right),$$

where P(·) denotes the patch unfolding operation, and $X_{p}$ represents the unfolded local patch features.

Then, a two-dimensional Fast Fourier Transform (2D-FFT) is applied to each local patch:

(18) $$X_{f}=F_{2D}\left(X_{p}\right),$$

where $F_{2D}\left(\cdot \right)$ denotes the two-dimensional Fourier transform, and $X_{f}$ represents the complex-valued frequency-domain representation of local patch features.

To explicitly model the importance of different frequency components, a learnable frequency-weight matrix $W_{f}$ is introduced to adaptively reweight the spectral responses:

(19) $${\hat{X}}_{f}=W_{f}⊙X_{f}.$$

where $W_{f}$ denotes the learnable frequency modulation matrix, and ${\hat{X}}_{f}$ denotes the enhanced frequency-domain feature. This adaptive reweighting mechanism enables the network to automatically emphasize frequency components that are more relevant to edge, texture, and structural restoration, while suppressing less informative or redundant frequency responses.

Finally, the enhanced frequency-domain feature is transformed back to the spatial domain through the inverse Fourier transform, and the local patches are reassembled to recover the original feature layout:

(20) $$\hat{X}=P^{-1}\left(F_{2D}^{-1}\left({\hat{X}}_{f}\right)\right),$$

where $F_{2D}^{-1}\left(\cdot \right)$ and $P^{-1}\left(\cdot \right)$ denote the inverse two-dimensional Fourier transform and patch folding operation, respectively. $\hat{X}$ denotes the reconstructed spatial-domain feature after frequency-aware enhancement.

Through the above process, FDEB forms a compact three-stage enhancement pipeline, namely, spatial projection and gating, block-wise frequency modulation, and spatial reconstruction. This design preserves the computational efficiency of FFT-based operations while enabling explicit discriminative modeling of frequency information. Compared with directly introducing complex attention-based local enhancement modules, FDEB provides a more efficient and controllable solution for improving local texture reconstruction and structural detail restoration, making it particularly suitable for lightweight image dehazing frameworks.

#### 3.3.4. Frequency-Aware State Interaction Block (FASI Block)

To further integrate the advantages of state propagation and frequency-aware enhancement, we propose a Frequency-Aware State Interaction Block (FASI Block). This module is designed to couple the FFT-Based State Space Block (FFTSSB) and the Frequency-Aware Discriminative Enhancement Block (FDEB) within a unified residual interaction framework, so as to achieve collaborative modeling of global structural dependencies and local discriminative details.

In UAV image dehazing, haze degradation not only reduces global contrast and structural consistency over large spatial regions, but also causes local blurring of edges and textures, leading to the loss of high-frequency details. As analyzed above, FFTSSB mainly focuses on long-range dependency modeling through bidirectional non-causal state propagation and is effective in enhancing global structural consistency and cross-position information integration. In contrast, FDEB emphasizes local discriminative enhancement in the frequency domain, adaptively modulating key frequency responses associated with edge, texture, and structural restoration. Since these two modules operate at different modeling levels and focus on different feature properties, directly using either one alone is insufficient for fully exploiting their complementary strengths.

Specifically, if only global state propagation is employed, the fine-grained detail enhancement introduced by local frequency modeling may be weakened during feature integration. Conversely, if only local frequency enhancement is considered without global structural constraints, the coordination of spatial information may become inadequate, which can affect structural continuity and restoration stability. Moreover, in a multi-scale encoder–decoder architecture, different feature resolutions correspond to different restoration objectives. Therefore, a unified interaction mechanism is required to facilitate effective collaboration between global state modeling and local frequency-aware enhancement across different scales.

Given an input feature representation $X\in R^{H\times W\times C}$, FASI first feeds the normalized feature into FFTSSB to perform bidirectional non-causal state propagation, and then fuses the resulting feature with the input through a residual connection:

(21) $$X_{s}=X+\mathrm{FFTSSB}\left(\mathrm{Norm}\left(X\right)\right),$$

where Norm(·) denotes the normalization operation, and $X_{s}$ denotes the state-enhanced feature. This branch mainly models long-range spatial dependencies and imposes global structural consistency constraints on the feature representation.

Based on the state-enhanced feature $X_{s}$, FASI further introduces FDEB to perform frequency-aware discriminative enhancement. Specifically, the normalized feature is fed into FDEB, and the enhanced result is added back through another residual connection:

(22) $$X_{\mathrm{out}}=X_{s}+\mathrm{FDEB}\left(\mathrm{Norm}\left(X_{s}\right)\right).$$

where $X_{\mathrm{out}}$ denotes the output feature of the FASI block. Here, FDEB explicitly emphasizes frequency components closely related to image dehazing, such as edges, textures, and contrast-sensitive structures, thereby complementing the global state propagation branch with stronger local detail restoration capability.

Through this serial residual interaction design, FASI forms a progressive feature refinement process of “state propagation enhancement–frequency discriminative enhancement”. Such a design preserves the global consistency modeling capability of state propagation while further improving local structural recovery through frequency-aware enhancement. At the same time, the two residual connections help alleviate optimization difficulty in deep networks, promote stable information transmission during repeated interaction, and preserve the original structural information of the input feature.

Structurally, FASI first performs feature normalization, then conducts global state propagation modeling and local frequency-aware enhancement through the FFTSSB and FDEB branches, respectively, and finally achieves unified feature fusion under residual constraints. In this way, the block is able to maintain the original structural basis of the input while progressively realizing the collaborative optimization of global consistency and local detail enhancement.

Furthermore, FASI is hierarchically embedded into multiple stages of the encoder–decoder architecture. As the feature resolution changes, FASI undertakes different but complementary modeling roles at different scales. In low-resolution encoder stages, FASI mainly focuses on global structure modeling and cross-region dependency enhancement; in high-resolution decoder stages, it places more emphasis on local detail recovery and edge continuity improvement; while in intermediate stages, it jointly balances global structure consistency and local texture optimization. Through this multi-scale embedding strategy, FASI continuously promotes the interaction between state propagation and frequency-aware enhancement throughout the network, enabling tighter collaboration among features at different resolutions and ultimately improving dehazing quality in complex UAV haze scenes.

Different from conventional frequency-domain modules that mainly perform spectral filtering or frequency reweighting, FASI introduces state space propagation into the spatial–frequency interaction process. FFTSSB captures long-range structural dependencies through FFT-based two-sided non-causal convolution, while FDEB restores haze-sensitive local textures and edges through frequency-aware enhancement. Compared with vanilla Mamba or SSM blocks that usually rely on directional scanning, FASI avoids one-directional information propagation and better matches the non-causal nature of image restoration. Therefore, FASI jointly improves global contextual consistency and local detail recovery, distinguishing it from CNN-based local modules, Transformer-based attention modules, conventional frequency-domain modules, and vanilla SSM/Mamba blocks.

### 3.4. Loss Function

The network is optimized by minimizing the following objective function:

(23) $$L=\|I_{\mathrm{dehaze}}-I_{\mathrm{gt}}{\|}_{1}+\lambda {∥F\left(I_{\mathrm{dehaze}}\right)-F\left(I_{\mathrm{gt}}\right)∥}_{1},$$

where $I_{\mathrm{dehaze}}$ and $I_{\mathrm{gt}}$ denote the restored dehazed image and the ground-truth clear image, respectively. F(·) denotes the discrete Fourier transform (DFT), ${\left\|\cdot \right\|}_{1}$ denotes the $L_{1}$ norm, and λ is a balancing coefficient empirically set to 0.1. The first term enforces pixel-wise spatial fidelity, while the second term encourages frequency-domain consistency, compensating for the limitations of purely spatial loss in preserving high-frequency structures and textures.

## 4. Experiments

### 4.1. Experimental Settings

#### 4.1.1. Dataset

To systematically evaluate the dehazing performance and generalization capability of the proposed FSSM (Frequency-Enhanced State Space Model) under complex haze conditions from UAV viewpoints, experiments are conducted on the HazyDet dataset [47]. HazyDet is a challenging UAV-oriented haze dataset that contains both synthetic and real hazy images, covering diverse haze densities, illumination conditions, and complex urban backgrounds. The dataset provides annotations for approximately 383,000 object instances, exhibiting high scene diversity and considerable difficulty.

In this work, paired hazy images and their corresponding ground-truth clear images are utilized for supervised dehazing training and evaluation. The dataset is split into training and testing sets with a ratio of 8:2. Moreover, the training and testing subsets are constructed to be scene-disjoint, ensuring an objective evaluation of model generalization performance.

It should be noted that this study mainly focuses on UAV-view image dehazing. Therefore, HazyDet is selected as the primary benchmark because it contains UAV-oriented hazy scenes with diverse haze densities, illumination conditions, and complex urban backgrounds, which are consistent with the application scenario considered in this work. Although additional commonly used dehazing benchmarks, such as RESIDE/SOTS and NH-HAZE, are valuable for broader cross-dataset evaluation, they differ from HazyDet in scene viewpoint, data collection conditions, and haze degradation characteristics. In this study, we focus on validating the effectiveness of FSSM under UAV-view haze degradation. More extensive multi-dataset evaluation will be considered in future work to further examine the cross-domain generalization capability and robustness of the proposed framework.

#### 4.1.2. Comparative Methods

To comprehensively evaluate the effectiveness of the proposed FSSM framework, a set of representative image-dehazing methods is selected for comparison, including traditional prior-based approaches and deep learning-based methods with different architectural characteristics. Specifically, the compared methods include DCP [4], AOD-Net [24], GridDehaze-Net [8], FFA-Net [25], MixDehaze-Net [26], OK-Net [27], and DWTMA-Net [28].

These methods cover a wide range of dehazing strategies, from physics-driven priors and early CNN-based dehazing networks to recent deep learning methods with multi-scale, attention-based, and frequency-aware designs. In particular, MixDehaze-Net, OK-Net, and DWTMA-Net are included as recent comparison methods to provide an up-to-date benchmark. Meanwhile, representative Transformer-based and SSM-based restoration methods, such as DehazeFormer, Restormer, UVM-Net, and Mamba-style image restoration networks, are discussed and analyzed in Section 2 to clarify the methodological differences between the proposed FSSM and recent long-range dependency modeling methods. All comparative methods included in the experiments are trained and evaluated under the same dataset split and hardware environment to ensure fair and consistent comparisons.

#### 4.1.3. Implementation Details

During data preprocessing, paired images from the HazyDet dataset are used for training and validation. Input images are cropped into patches of size 128×128, followed by random horizontal flipping and rotation for data augmentation. All input images are normalized to the range [0,1] before being fed into the network.

The proposed FSSM adopts a three-level encoder–decoder architecture, with channel dimensions set to 48, 96, and 192, respectively. At the three corresponding scales, [6, 6, 12] Frequency-Aware State Interaction (FASI) blocks are stacked, and the feed-forward expansion ratio is set to 3. The network is optimized using the AdamW optimizer with momentum parameters $\beta_{1}=0.9$ and $\beta_{2}=0.9$. A cosine annealing learning rate scheduler is employed, with the initial learning rate set to $2\times 10^{-4}$ and gradually decayed to $1\times 10^{-6}$ over 300,000 iterations. No warm-up strategy is applied during training.

#### 4.1.4. Evaluation Metrics

To quantitatively evaluate dehazing performance, three widely used evaluation metrics are adopted:

- PSNR (Peak Signal-to-Noise Ratio): measures the pixel-level reconstruction accuracy between the dehazed image and the ground-truth image;
- SSIM (Structural Similarity Index): evaluates the consistency of structural information;
- NIQE (Natural Image Quality Evaluator): reflects the perceptual naturalness of images, where lower values indicate better visual quality.

In addition to PSNR, SSIM, and NIQE, model complexity is also evaluated to assess the practical efficiency of different dehazing methods. Specifically, FLOPs and parameter count are reported to measure computational cost and model size, respectively. Since the proposed FSSM is motivated by efficient long-range dependency modeling, these complexity metrics provide important evidence for evaluating whether the proposed FFT-based State-Space Modeling strategy can balance dehazing performance and computational efficiency.

### 4.2. Quantitative Evaluations

The quantitative comparison results of different dehazing methods on the HazyDet dataset are summarized in Table 1.

PSNR, SSIM, and NIQE are employed to comprehensively evaluate dehazing performance from the perspectives of pixel-level reconstruction accuracy, structural consistency, and perceptual image quality, respectively.

As shown in Table 1, the proposed FSSM achieves the highest PSNR (29.97 dB) and SSIM (0.9396) among all compared methods, while also obtaining the lowest NIQE score (11.20). These results indicate that FSSM exhibits strong overall advantages in detail restoration, structural preservation, and perceptual naturalness. Compared with traditional prior-based methods such as DCP and early deep learning approaches like AOD-Net, FSSM achieves substantial improvements across all three metrics, demonstrating its effectiveness in addressing complex haze degradation in UAV-view scenarios.

When compared with CNN-based dehazing methods such as GridDehaze-Net and FFA-Net, FSSM consistently yields higher PSNR and SSIM values and achieves superior NIQE performance, suggesting that it is more capable of preserving natural image appearance while removing haze. Furthermore, compared with recently proposed methods including MixDehaze-Net, OK-Net, and DWTMA-Net, FSSM maintains a clear advantage in overall quantitative performance, highlighting its robustness and stability in challenging UAV haze scenes.

Overall, these quantitative results demonstrate that by incorporating frequency-enhanced State-Space Modeling, FSSM effectively improves the modeling of long-range dependencies and frequency-correlated features, leading to superior dehazing performance on the HazyDet dataset.

### 4.3. Computational Complexity Analysis

To further evaluate the computational efficiency of the proposed method, we compare the FLOPs and parameter count of different dehazing methods, as shown in Table 2. FLOPs reflect the computational cost of the model during inference, while the number of parameters indicates the model size and storage requirement. These two metrics provide a quantitative basis for evaluating the practical deployment potential of different dehazing models.

As shown in Table 2, different dehazing methods exhibit obvious differences in computational complexity. Traditional prior-based methods such as DCP do not involve learnable parameters, while deep learning-based methods introduce different levels of computational costs. The proposed FSSM contains 16.74 M parameters and requires 246.66 G FLOPs. Although its parameter count is larger than that of the compared CNN-based methods, its FLOPs are lower than those of FFA-Net and comparable to those of several recent high-performance dehazing networks.

Combining Table 1 and Table 2, FSSM achieves the best PSNR, SSIM, and NIQE results among all compared methods, while maintaining a moderate computational cost compared with high-complexity dehazing networks such as FFA-Net. This indicates that the performance improvement of FSSM is not only achieved by increasing model capacity, but also benefits from the proposed frequency-enhanced State-Space Modeling strategy. In particular, the FFT-based state space block improves long-range dependency modeling without relying on dense self-attention over high-resolution feature maps.

The efficiency of FSSM mainly benefits from the FFT-based implementation of state-space convolution. Instead of using explicit recursive state updates or dense self-attention, FFTSSB performs global feature interaction in the frequency domain. This design enables the model to capture long-range dependencies with an efficient convolutional form. Therefore, the proposed method provides a practical trade-off between restoration quality and computational complexity for UAV-view image dehazing tasks that require global structural recovery and detail preservation.

### 4.4. Qualitative Evaluations

**Figure 2:**
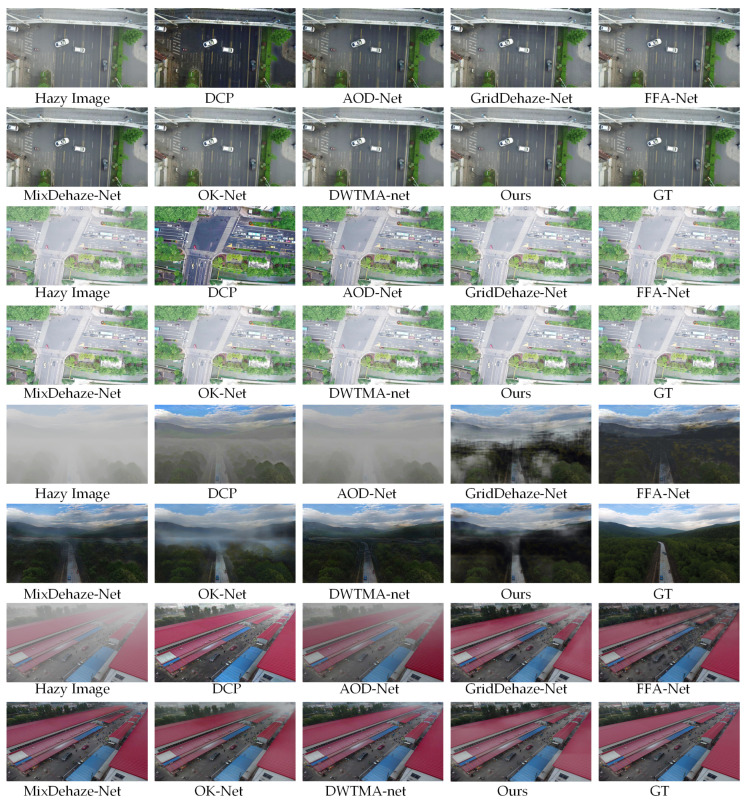
Qualitative comparison of different dehazing methods on the HazyDet dataset.

To further provide an intuitive assessment of the dehazing performance of different methods under real UAV haze scenarios, several representative test samples from the HazyDet dataset are selected for qualitative comparison. The visual comparison results are shown in Figure 2.

As illustrated in Figure 2, traditional prior-based methods such as DCP tend to suffer from noticeable over-enhancement and color distortion in certain regions. Some CNN-based approaches, including AOD-Net and GridDehaze-Net, are able to remove a large portion of haze; however, residual haze and blurred details can still be observed in distant regions.

In contrast, the proposed FSSM demonstrates more stable and consistent restoration performance, particularly in sky regions, road boundaries, and long-range structures. FSSM effectively removes haze while preserving natural color distributions and clear structural details. Notably, in UAV scenes with varying haze density and complex backgrounds, FSSM shows superior capability in suppressing residual haze, enhancing local contrast, and maintaining structural consistency. These qualitative observations are highly consistent with the quantitative evaluation results, further validating the effectiveness of the proposed frequency-enhanced State Space Modeling framework.

The visual comparison further focuses on representative UAV-view regions, including distant structures, sky areas, road boundaries, and fine texture regions. Compared with the competing methods, FSSM better suppresses residual haze in distant regions and maintains a more natural color appearance in large sky areas. Meanwhile, it preserves clearer road boundaries and local structural details, reducing over-smoothed artifacts commonly observed in some CNN-based dehazing results. These visual improvements indicate that the proposed FDEB contributes to local detail preservation, while FFTSSB enhances long-range structural consistency. Overall, the qualitative results further support the quantitative improvements reported in Table 1.

### 4.5. Ablation Study

To further evaluate the effectiveness and necessity of the key components in the proposed framework, ablation studies are conducted on the two core modules of FSSM, namely the FFT-Based State Space Block (FFTSSB) and the Frequency-Aware Discriminative Enhancement Block (FDEB). These two modules directly correspond to the main design motivations of the proposed method: global dependency modeling through FFT-based state space propagation and local detail restoration through frequency-aware discriminative enhancement. All ablation experiments are carried out under the same training strategy, dataset split, and testing settings. Only the corresponding modules are replaced or removed to ensure fair comparison. PSNR and SSIM are adopted as the evaluation metrics, and the test set contains 2000 images.

It should be noted that the Frequency-Aware State Interaction (FASI) block is constructed by integrating FFTSSB and FDEB within a unified residual interaction framework. Therefore, the current ablation design mainly verifies the effectiveness of the two key functional branches inside FASI. The FFTSSB ablation evaluates the contribution of FFT-based State Space Modeling to global structural consistency, while the FDEB ablation evaluates the contribution of frequency-aware local enhancement to texture and edge restoration. The present study focuses on isolating the newly designed modules, namely FFTSSB and FDEB, because they directly correspond to the main technical contributions of the proposed FSSM. This ablation design provides direct evidence for verifying the effectiveness of the proposed core modules.

#### 4.5.1. Ablation Study on FFTSSB

The FFTSSB serves as a core component of the proposed model for global dependency modeling and frequency-domain state propagation. To evaluate its effectiveness, FFTSSB is replaced with two commonly used attention mechanisms, namely standard self-attention and linear attention, while keeping all other components unchanged. The quantitative comparison results are reported in Table 3.

As reported in Table 3, the proposed method achieves the best performance, with PSNR and SSIM reaching 29.9695 dB and 0.9396, respectively. Replacing FFTSSB with standard self-attention results in a slight PSNR decrease of 0.0445 dB, while SSIM drops more noticeably to 0.9236. These results indicate that although self-attention can achieve comparable haze removal in terms of pixel-level error, it is less effective in preserving structural consistency and fine-grained details.

Furthermore, replacing FFTSSB with linear attention results in a more pronounced performance degradation, with PSNR and SSIM decreasing to 29.0133 dB and 0.9295, respectively. This suggests that while linear attention is computationally efficient, its ability to model global dependencies and frequency-related structures is limited in the image-dehazing task. Overall, these results demonstrate that FFTSSB is more suitable for image dehazing, as it effectively balances global modeling capability and structural consistency.

#### 4.5.2. Ablation Study on FDEB

The Frequency-Aware Discriminative Enhancement Block (FDEB) integrates spatial gated convolution with FFT-based frequency modeling to achieve joint spatial–frequency feature enhancement. To quantitatively evaluate the contribution of each component in FDEB, two ablation experiments are conducted, and the corresponding results are summarized in Table 4.

As shown in Table 4, when the FFT frequency branch in FDEB is removed while retaining the spatial gated feed-forward structure, the PSNR and SSIM decrease to 29.7928 dB and 0.9389, respectively. This result indicates that although the spatial gated FFN is capable of modeling local structures to a certain extent, the absence of frequency-domain modeling leads to performance degradation in fine-detail restoration and structural consistency, highlighting the importance of the FFT branch for selective frequency recovery.

Furthermore, when FDEB is completely replaced by a standard feed-forward network (FFN/MLP), the performance further degrades, with PSNR and SSIM dropping to 29.2409 dB and 0.9365, respectively. This observation demonstrates that the performance gains of FDEB are not solely attributed to the frequency-domain branch, but also benefit from the joint design of spatial gating and frequency-aware enhancement. Compared with conventional FFN structures, FDEB more effectively captures complex spatial–frequency characteristics under haze degradation, thereby improving local texture recovery and structural fidelity.

#### 4.5.3. Qualitative Analysis of Ablation Results

**Figure 3:**
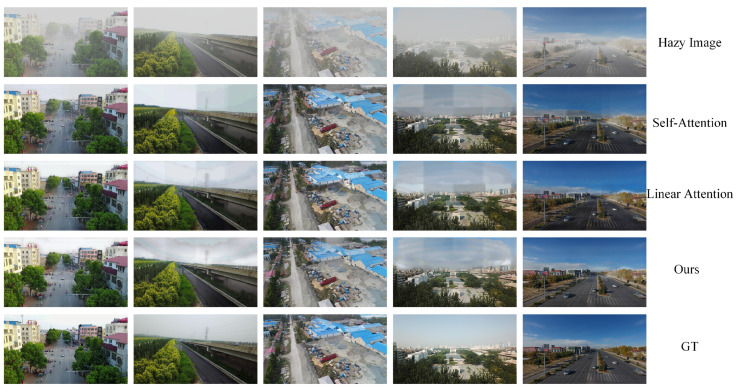
Qualitative ablation results of the FFT-based State-Space Block (FFTSSB).

**Figure 4:**
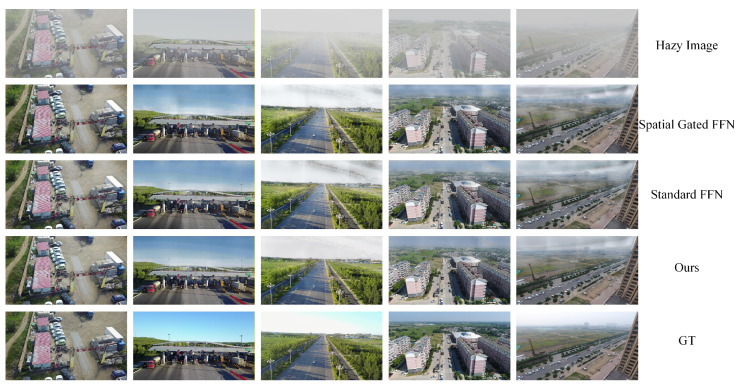
Qualitative ablation results of the Frequency-Aware Discriminative Enhancement Block (FDEB).

In addition to quantitative evaluations, qualitative comparisons under different ablation configurations are further conducted, as illustrated in Figure 3 and Figure 4.

As shown in Figure 3, in the ablation study of the FFT-based State Space Block (FFTSSB), the complete model effectively suppresses residual haze in sky regions, long-range structures, and object boundaries, while preserving clear and stable structural details. In contrast, when FFTSSB is replaced by Self-Attention or Linear Attention, the dehazing results exhibit varying degrees of residual haze, structural blurring, and insufficient contrast, particularly in distant regions. These observations indicate the limitations of conventional attention mechanisms in global dependency modeling and structural consistency under complex UAV haze conditions.

As shown in Figure 4, in the ablation study of the Frequency-Aware Discriminative Enhancement Block (FDEB), removing the frequency-domain branch or replacing FDEB with a standard feed-forward network (FFN) leads to noticeable degradation in detail restoration, characterized by blurred textures and weakened edge structures. By comparison, the complete FDEB achieves more stable enhancement of local contrast and finer structural details, especially in complex backgrounds and low-contrast regions.

Overall, these qualitative observations are highly consistent with the quantitative evaluation trends, further validating the critical role of FFTSSB in global dependency modeling and the effectiveness of FDEB in fine-grained detail enhancement and structural restoration.

## 5. Conclusions

In this paper, we addressed the challenges of insufficient global dependency modeling and limited computational efficiency in image dehazing under complex UAV-view haze scenarios. To this end, we proposed an efficient frequency-enhanced State Space Modeling framework, termed FSSM (Frequency-Enhanced State Space Model). By adopting State Space Modeling as the overall architectural backbone and introducing FFT-based two-sided non-causal convolution, FSSM enables efficient global dependency modeling while reducing the computational burden caused by explicit recursive scanning and dense self-attention, thereby achieving a favorable balance between dehazing performance and inference efficiency.

From a methodological perspective, we developed a novel FFT-based State Space Block (FFTSSB), which replaces conventional recursive scanning mechanisms with implicit state propagation in the frequency domain, effectively alleviating the limitations of unidirectional modeling and computational redundancy. Furthermore, we introduced a Frequency-Aware State Interaction module (FASI), which integrates FFTSSB with the Frequency-Aware Discriminative Enhancement Block (FDEB). This design facilitates collaborative optimization of global structural consistency and local detail restoration in both spatial and frequency domains. Based on these core components, a multi-scale hierarchical encoder–decoder architecture was constructed to enhance the modeling capability for complex haze degradation patterns.

Experimental results on the HazyDet dataset demonstrate that the proposed FSSM achieves favorable performance in terms of PSNR, SSIM, and NIQE, while also producing more stable and visually pleasing results in real UAV haze scenes. Ablation studies further verify the important roles of FFTSSB and FDEB in improving global structural consistency and local detail restoration, confirming the effectiveness of the proposed framework for challenging image-dehazing tasks.

Despite the promising performance of FSSM, there remains room for further improvement. First, the current framework is primarily designed for single-image dehazing and does not explicitly incorporate temporal consistency constraints. Second, although HazyDet provides diverse UAV-view hazy scenes, more extensive evaluations on commonly used dehazing benchmarks, such as RESIDE/SOTS and NH-HAZE, would further strengthen the analysis of cross-domain generalization. Third, although the proposed method has been evaluated in terms of PSNR, SSIM, NIQE, FLOPs, and parameter count, additional deployment-oriented indicators, such as inference latency, memory consumption, and FPS on edge devices, can provide more comprehensive evidence for practical deployment. Future work will explore extending FSSM to video dehazing and multi-weather image restoration tasks, as well as optimizing the inference pipeline for practical edge-device deployment.

Overall, the proposed FSSM provides an efficient and interpretable solution for applying State Space Models to image dehazing, offering useful insights and a practical foundation for UAV visual perception in complex real-world environments.

## Figures and Tables

**Table 1:** Quantitative comparison on the HazyDet dataset, where the best results are highlighted in bold.

Methods	Publication	PSNR (dB)	SSIM	NIQE
DCP	TPAMI 2011	17.03	0.8024	12.30
AOD-Net	ICCV 2017	18.99	0.7808	12.27
GridDehaze-Net	ICCV 2019	26.66	0.8801	11.33
FFA-Net	AAAI 2020	27.12	0.8782	11.31
MixDehaze-Net	IJCNN 2024	28.75	0.9068	11.30
OK-Net	AAAI 2024	27.76	0.8875	11.36
DWTMA-Net	Sensors 2025	29.02	0.9108	11.23
**Ours**	–	**29.97**	**0.9396**	**11.20**

**Table 2:** Comparison of computational complexity among different dehazing methods.

Method	FLOPs	Parameters
DCP	–	–
AOD-Net	457.70 M	1.76 K
GridDehaze-Net	85.72 G	955.75 K
FFA-Net	624.20 G	4.68 M
MixDehaze-Net	114.30 G	3.17 M
OK-Net	158.20 G	4.43 M
DWTMA-Net	188.72 G	8.34 M
**Ours**	246.66 G	16.74 M

**Table 3:** Comparison of ablation results for FFTSSB.

Method	Global Modeling Module	PSNR (dB)	SSIM
Linear Attention	Linear Attention	29.0133	0.9295
Multi-Head Attention	Self-Attention (MHSA)	29.9250	0.9236
Proposed Method	FFTSSB	29.9695	0.9396

**Table 4:** Comparison of ablation results for FDEB.

Method	FFT Branch	FFN Type	PSNR (dB)	SSIM
w/o FFT Branch	×	Spatial Gated FFN	29.7928	0.9389
Replace FDEB with Standard FFN	×	Standard FFN (MLP)	29.2409	0.9365
Proposed Method	✓	FDEB	29.9695	0.9396

×: Not included; ✓: Included.

## Data Availability

The HazyDet dataset used in this study is publicly available at https://github.com/GrokCV/HazyDet, accessed on 19 April 2026. All experimental results and code are available from the corresponding author upon reasonable request.
